# Establishment and genomic characterizations of patient-derived esophageal squamous cell carcinoma xenograft models using biopsies for treatment optimization

**DOI:** 10.1186/s12967-018-1379-9

**Published:** 2018-01-25

**Authors:** Jianling Zou, Ying Liu, Jingyuan Wang, Zhentao Liu, Zhihao Lu, Zuhua Chen, Zhongwu Li, Bin Dong, Wenwen Huang, Yanyan Li, Jing Gao, Lin Shen

**Affiliations:** 1 Department of Gastrointestinal Oncology, Key Laboratory of Carcinogenesis and Translational Research (Ministry of Education), Peking University Cancer Hospital & Institute, Fu-Cheng Road 52, Hai-Dian District, Beijing, 100142 China; 2 Laboratory of Genetics, Key Laboratory of Carcinogenesis and Translational Research (Ministry of Education), Peking University Cancer Hospital & Institute, Beijing, 100142 China; 3 Department of Pathology, Key Laboratory of Carcinogenesis and Translational Research (Ministry of Education), Peking University Cancer Hospital & Institute, Beijing, China

**Keywords:** Patient-derived xenograft model, Esophageal squamous cell carcinoma, Biopsy, Genomic characterizations

## Abstract

**Background:**

Squamous cell carcinoma is the dominant type of esophageal cancer in China with many patients initially diagnosed at advanced stage. Patient-derived xenografts (PDX) models have been developed to be an important platform for preclinical research. This study aims to establish and characterize PDX models using biopsy tissue from advanced esophageal cancer patients to lay the foundation of preclinical application.

**Methods:**

Fresh endoscopic biopsy tissues were harvested from patients with advanced esophageal cancer and implanted subcutaneously into NOD/SCID mice. Then, the PDXs were serially passaged for up to four generations. Transplantation was analyzed and genomic characteristics of xenografts were profiled using next-generation sequencing.

**Results:**

Twenty-five PDX models were established (13.3%, 25/188). The latency period was 75.12 ± 19.87 days (50–120 days) for the first passage and it decreased with increasing passaging. Other than tumor stages, no differences were found between transplantations of xenografts and patient characteristics, irrespective of chemotherapy. Histopathological features and chemosensitivity of PDXs were in great accordance with primary patient tumors. Each PDX was assessed for molecular characteristics including copy number variations, somatic mutations, and signaling pathway abnormalities and these were similar to patient results.

**Conclusions:**

Our PDX models were established from real time biopsies and molecularly profiled. They might be promising for drug development and individualized therapy.

**Electronic supplementary material:**

The online version of this article (10.1186/s12967-018-1379-9) contains supplementary material, which is available to authorized users.

## Background

Esophageal cancer is the third most common cancer and the fourth leading cause of cancer death in all cancer types in China [1]. Esophageal squamous cell carcinoma (ESCC) is the predominant histological type, representing more than 95% of all cases in China [2, 3]. Most patients are initially diagnosed at advanced stages and cannot be surgically removed. At this time, chemotherapy and radiotherapy are primary approaches for treatment but this offers little benefit: five-year survival has not changed in decades [4, 5]. Due to the limited therapeutic agent available and frequent drug resistance, it is urgent to exploit new agents and explore the mechanisms of drug resistance [6].

Recent studies indicate that various tumorigenic signaling pathways are involved in ESCC, such as tumor growth, cell cycle, angiogenesis, invasion and apoptosis [7–9], so targeting these signaling pathways may be strategies for treating ESCC. Target therapy also showed exciting effect when combined with immunotherapy [10–12]. However, there is no specific agent for targeted therapy in ESCC. Therefore it is urgent to identify promising targets for therapy to improve the survival of ESCC patients.

Recently, patient-derived xenograft (PDX) models have been developed for translating basic research into clinical solutions and these offer advantages over cell line-based model [13, 14]. Because PDX involves transplanting cancer patients tissue directly into immunocompromised mice, genetic information, immunohistological markers and chemosensitivity are correlative to the patient and can be applied to evaluate new antitumor drugs [6, 13, 15, 16]. Thus PDX models provide an irreplaceable platform to study biological and genetic alterations, as well as potential anticancer therapies.

Little literature exists to describe PDX models of ESCC, and most were established using surgical tissues with relatively earlier tumor stages [17–21]. However, patients with advanced staged tumors are better for evaluating the efficacy of new agents, and real-time endoscopic biopsy is the primary way to obtain tumor tissues in daily clinical works. Thus, PDX models generated with endoscopic biopsies from advanced patients may be more useful for drug development and guiding individualized therapy.

In this study, we established PDX models of ESCC with endoscopic biopsy tissues successfully and assessed the clinical and pathological factors associated with engraftment as well as the chemosensitivity of xenograft. Finally, genomic characterizations of PDXs were identified to explore new agents of targeted therapy in ESCC.

## Methods

### Patients and tissue samples

The research proposal had been approved by the Medical Ethics Committee of Peking University Cancer Hospital according to principles of the Declaration of Helsinki. Written informed consent was obtained from all study participants for their information to be stored in the hospital database and used for future research at the time of follow-up ascertainment. All the patients with pathologically confirmed esophageal carcinoma and available endoscopic biopsied samples were included in the study.

### Establishment of PDX models

NOD/SCID mice (6 weeks) were from Beijing Vital River Laboratory Animal Technology Co., Ltd. Two tissue fragments (~ 2 × 2 × 2 mm^3^/fragment) from fresh biopsies were obtained from each patient (P0 = passage zero), and were subcutaneously implanted into the flank of each mouse under sterile conditions. Tumor growth was assessed by palpation or Vernier calipers twice weekly. The established PDX model was passage 1 (P1). Mice were euthanized and harvested fresh tumor fragments were re-implanted into other mice when P1 tumors reached ~ 750 mm^3^. If animals showed disease, the tumor was collected when palpable. Subsequent passages were P2, P3, and P4. All procedures were performed under sterile conditions at Peking University Cancer Hospital specified-pathogens free facility and carried out in accordance with the Guide for the Care and Use of Laboratory Animals of the NIH. Each model derived from individual patients was passaged for up to four generations. At each passage, tumor tissues were cryopreserved (90% FBS and 10% DMSO), snap-frozen in liquid nitrogen for future use, and fixed in neutral buffered formalin for histological examination.

### RNA extraction and quantitative real-time PCR

Total RNA was extracted from fresh PDX samples with TRIzol reagent (Invitrogen) in accordance with the manufacturer’s protocol. RNA concentration was quantified using a Nanodrop (Thermo Scientific, Hemel Hempstead, UK) and diluted to 100 ng/ml in RNase-free water before using the TransScript II One-Step gDNA Removal and cDNA Synthesis SuperMix kit (TransGen Biotech) according to the introduction. The cDNA was kept at − 20 °C until used for qPCR. Quantitative real-time RT-PCR analyses were performed using the SYBR^®^ Green Realtime PCR Master Mix (TOYOBO, BIOTECH CO., LTD), the BIO-RAD CFX96TM Real-Time System and Bio-Rad CFX Manager 2.1 software (Bio-Rad Laboratories, Inc., USA). The primer sequences for human FGF3 were: forward, 5′-ATGCTTCGGAGCACTACAGC-3′, and reverse, 5′-CCGTTCACAGACACGTACCA-3′. The primer sequences for human FGF4 were: forward, 5′-CTATGGCTCGCCCTTCTTCA-3′, and reverse, 5′-CCATTCTTGCTCAGGGCGAT-3′. The primer sequences for human FGF19 were: forward, 5′-AGATCAAGGCAGTCGCTCTG-3′, and reverse, 5′-GAGTACTGAAGCAGCCCCTG-3′. The primer sequences for human GAPDH were: forward, 5′-TTTGGTATCGTGGAAGGACT-3′, and reverse, 5′-AGTAGAGGCAGGGATGATGT-3′. The reaction conditions were an initial step of 95 °C for 60 s, followed by 40 cycles of denaturation at 95 °C for 15 s and annealing for 15 s, and extension at 72 °C for 45 s. Expression was normalized for RNA loading using GAPDH primers designed to span an intron and relative expression in each sample was calculated. To compare the difference of mRNA expression, fold changes were calculated by dividing the samples into two groups: CNV ≥ 5 or CNV < 5. Results are expressed as means ± SEMs. The semi-quantitative RT-PCR analyses from one individual experiment were repeated three times with comparable results.

### H&E staining

Histopathology of primary P0 tumors and xenografts were evaluated using H&E staining according to standard method with an H&E staining kit (C0105, Beyotime, China). Results were reviewed by two independent pathologists.

### Chemosensitivity of PDX model

Patient-derived xenografts models with 3 or more passages were used to evaluate chemosensitivity. Tumor bearing mice (~ 150 mm^3^ tumors) were randomized into two groups: treatment (paclitaxel, 10 mg/kg; platinum, 5 mg/kg, ip) and controls (saline). Treatment regimens were consistent with patient treatment and doses were determined from the literature [22–24]. Treatment was given once weekly for 3 cycles. Tumor size was measured twice weekly as described and tumor volume (V) was calculated as follows: V = L*W^2^/2 (L, length, long diameter of tumor; W, width, short diameter of tumor). All animal procedures were approved by the ethics committee for animal experiments at Peking University Cancer Hospital. Tumor growth inhibition (TGI) = (1 − ΔT/ΔC) × 100% (ΔT = tumor volume change of the drug-treated group on the final day of the study, ΔC = tumor volume change of the control group on the final day of the study).

### Targeted next-generation sequencing and data analysis

Genomic DNA was extracted from P4 xenografts of each PDX model using a QIAamp DNA Mini Kit (QIAGEN Ltd., Crawley, UK) according to the manufacturer’s instructions. Extracted DNA was evaluated using a Qubit fluorometer (Invitrogen, Carlsbad, CA) and 1% agarose gel electrophoresis. A custom 483 cancer-related gene panel was used (Additional file 1: Table S1) [25]. The capture-based library was generated from 500 ng of DNA from each sample using a KAPA Hyper Prep Kit according the manufacture’s instruction (Kapa Biosystems, Boston, MA, USA), followed by Agilent’s SureSelectXT Target Enrichment System (Agilent Technologies, Santa Clara, CA). Library quality was assessed using Agilent 2100 Bioanalyzed on-chip electrophoresis (Agilent Technologies, Inc), and the library was quantified with an Agilent QPCR NGS Library Quantification Kit (Agilent Technologies, Inc). The library was sequenced on an Illumina Hiseq 2000 system (Illumina, San Diego, CA).

Sequencing adapters and low quality reads were filtered and the final Q20 and Q30 of all samples were > 90 and > 85%, respectively. The BWA software with default parameters was used to align sequencing reads to the human reference hg19 genome, and Picard was used to mark duplications. The aligned reads achieved coverage of > 99% of the target region with a mapping rate of > 95%. The average sequencing depth was more than 1200 × per sample. Mutations were called using Samtools, Mutect, and Varscan software, and were annotated using Annovar software. CNV analysis was performed using Event-Wise Testing algorithm according to previous reports [26]. A neutral copy number for each exon for each gene was established using normal lymphocyte samples.

### Statistical analysis

Data were assessed for clinicopathological characteristics and transplantation success using a Chi squared test. An unpaired two-tailed Student’s *t* test was used to analyze the latency period of xenografts. Tumor growth between groups was compared using repeated-measured analysis of variance. A *p* value < 0.05 was considered statistically significant. Statistical analysis was performed with SPSS software for Windows, version 21 (SPSS Inc., Chicago, IL, USA).

## Results

### Establishment of PDX models and patient clinical characteristics

**Fig. 1:**
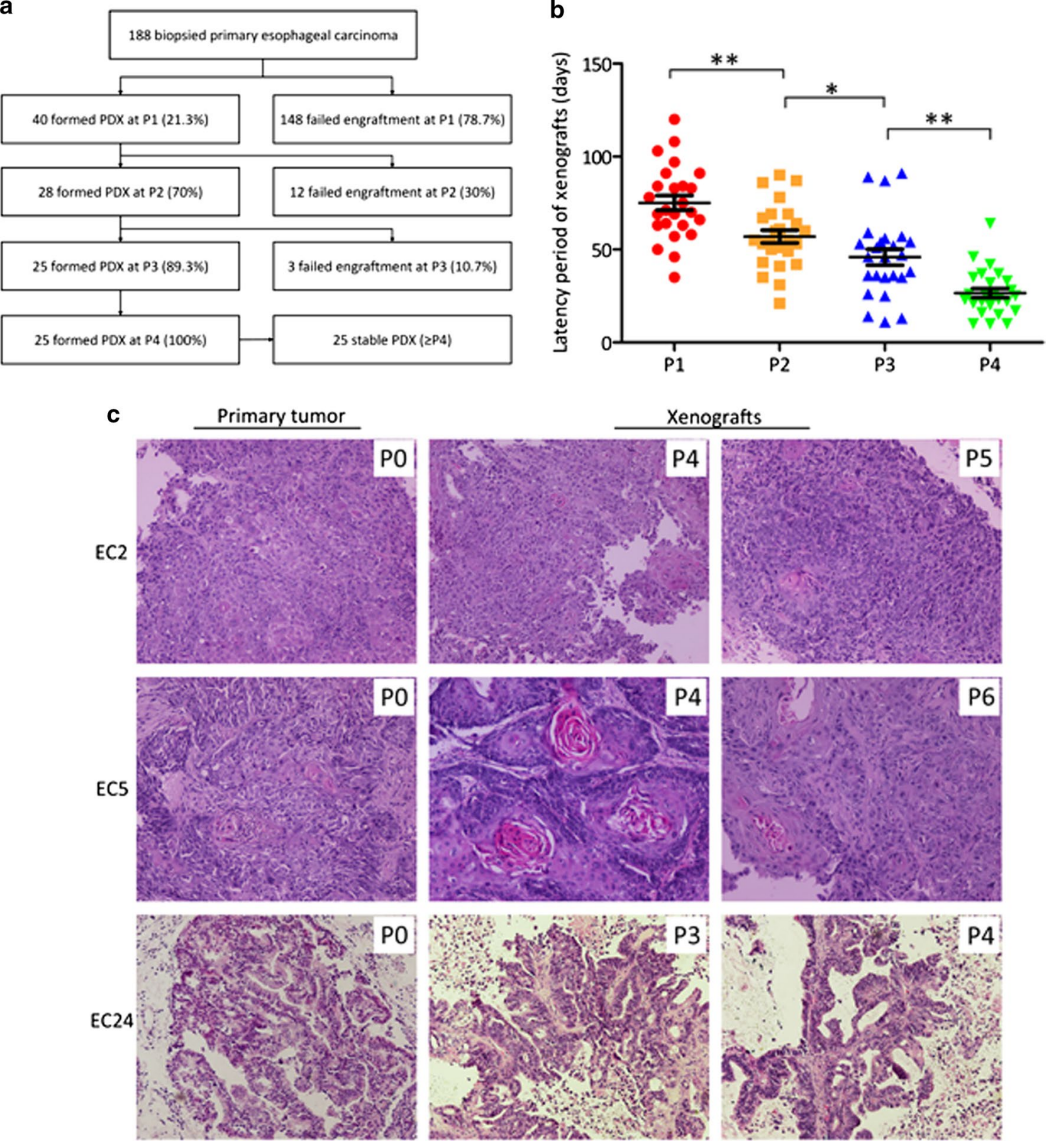
Establishment and histological characterization of PDX models from esophageal cancer patients. **a** Diagram of establishment of a stable PDX bank. **b** Decreasing latency with increasing passage. Line and error bars represent mean ± SD. *p* values were calculated using an unpaired two-tailed Student’s *t* test. *p < 0.05; **p < 0.01. **c** Representative histology of paired patient-PDX tumors. Slides were stained with H&E and images were captured at ×200

A total of 188 esophageal carcinoma samples were obtained by endoscopic biopsy, and 40 PDX models were established in NOD/SCID mice at P1 (Fig. 1a; transplantation success 21.3%) Along with serial passaging, the transplantation rates from P1 to P2, P2 to P3, P3 to P4 were 70% (28/40), 89.3% (25/28), 100% (25/25), respectively. After the fourth generation, the PDX models became stable without further change in model formation, which could be used in future study. The overall transplantation rate for PDX models in our study was 13.3% (25/188). The median latency period of xenografts was decreased along with serial passage with 75.12 ± 19.87 days, 56.96 ± 17.17 days, 45.88 ± 21.38 days, and 28.04 ± 16.23 days at P1, P2, P3, and P4 passage (Fig. 1b, *p* < 0.05). Due to tissue limitations for the first two passages, a bio-specimen bank of xenografts from passage three were established, including formalin fixed paraffin-embedded blocks, viably cryopreserved and snap frozen tissues. Cryopreserved xenografts could be re-grown in mice, providing a renewable tissue source for future usage.

No difference was observed between transplantation rates and patient characteristics except for tumor stage (Additional file 1: Table S2). Transplantation rates of biopsied samples from stage III or IV (17.7%, 22/124) was much higher than that from early stages (0, 0/19, *p* < 0.05). Table 1 shows data for the 25 PDX models established. All tissues were from males and from tumors located in median (44%) or lower (44%) esophagus. Histopathological and treatment data was shown in Table 1.

**Table 1:** Summary of characteristics of established PDX models for esophageal cancer (all male)

Case no.	Age	Tumor location	Differentiation	Stage	Treatment status
EC1	48	Lower	Poor	IV	Progressive disease
EC2	58	Median	Poor	IV	Before treatment
EC3	41	Lower	Moderate	IV	Before treatment
EC4	58	Lower	Poor	IV	Before treatment
EC5	51	Median	Moderate	IV	Before treatment
EC6	53	Median	Poor	IV	Before treatment
EC7	46	Lower	Moderate	III	Stable disease
EC8	49	Lower	Poor	IV	Before treatment
EC9	57	Median	Moderate	III	Before treatment
EC10	54	Median	Poor	IV	Before treatment
EC11	66	Lower	Poor	IV	Before treatment
EC12	49	Lower	Moderate	IV	Before treatment
EC13	68	Lower	Moderate	IV	Before treatment
EC14	55	Upper	Moderate	IV	Before treatment
EC15	63	NA	Moderate	NA	NA
EC16	62	Median	Poor	NA	NA
EC17	69	Upper	Moderate	IV	Before treatment
EC18	51	Median	Moderate	III	Before treatment
EC19	56	Median	Moderate	IV	Before treatment
EC20	79	Median	Moderate	IV	Before treatment
EC21	66	Lower	Moderate	IV	Stable disease
EC22	53	Lower	Poor	IV	Before treatment
EC23	59	Median	Poor	IV	Before treatment
EC24^a^	60	Median	Moderate	IV	Before treatment
EC25^a^	68	Lower	Moderate	NA	NA

All cases were of squamous carcinoma with the exception of those noted ^a^which were adenomas

*NA* non-available

### Histology of primary tumors and xenografts

Pathological assessment with H&E staining and analysis revealed consistent morphology and histology in xenografts compared with that of the corresponding primary tumor tissues (Fig. 1c; Additional file 1: Table S3). For case EC2, the PDXs kept poor differentiation from P1 to P5, which was in good accordance with the primary tumor. For case EC5, there was some variation in differentiation, with moderate differentiation in patient tumor and PDX of P1 to P6, except for P4, which was well differentiation (Fig. 1c). We then compared the histology type and differentiation of primary tumor and the two consecutive generation of P3, P4, and found that all the cases keep the same histology, and most cases kept the differentiation consistent (64%, 16/25), with some small changes in different passage (Additional file 1: Table S3).

### Sensitivity of PDX models to chemotherapy

**Fig. 2:**
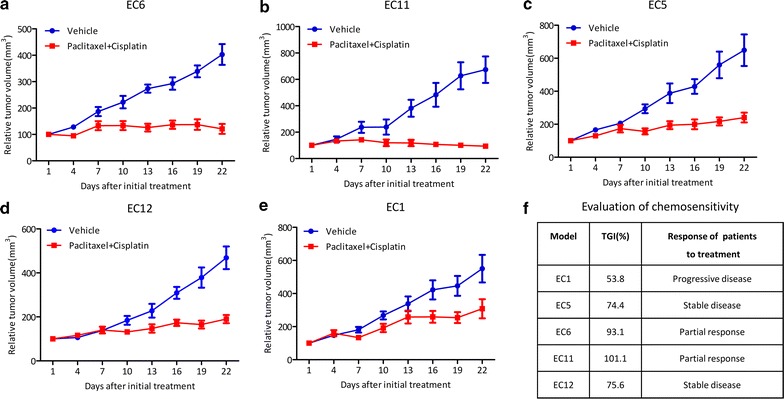
Therapeutic response of PDX models and corresponding patients. Five PDX models of ESCC were evaluated for therapeutic responses compared to corresponding patients. Better response was seen in PDX models with tumor tissue from patients with partial responses (**a**, **b**) compared to those with stable disease (**c**, **d**). Case EC1 (patient with progressive disease) had a minimum response (**e**). TGI and response for corresponding patients are given (**f**)

Five PDX models were treated with the clinically used chemotherapy agents, paclitaxel and cisplatin. Our data demonstrated that all of the PDX models had comparable therapeutic responses with that of the corresponding patients. The detailed data of the five PDX models and corresponding patients was shown in Fig. 2.

### Genomic characteristics of ESCC PDXs

**Fig. 3:**
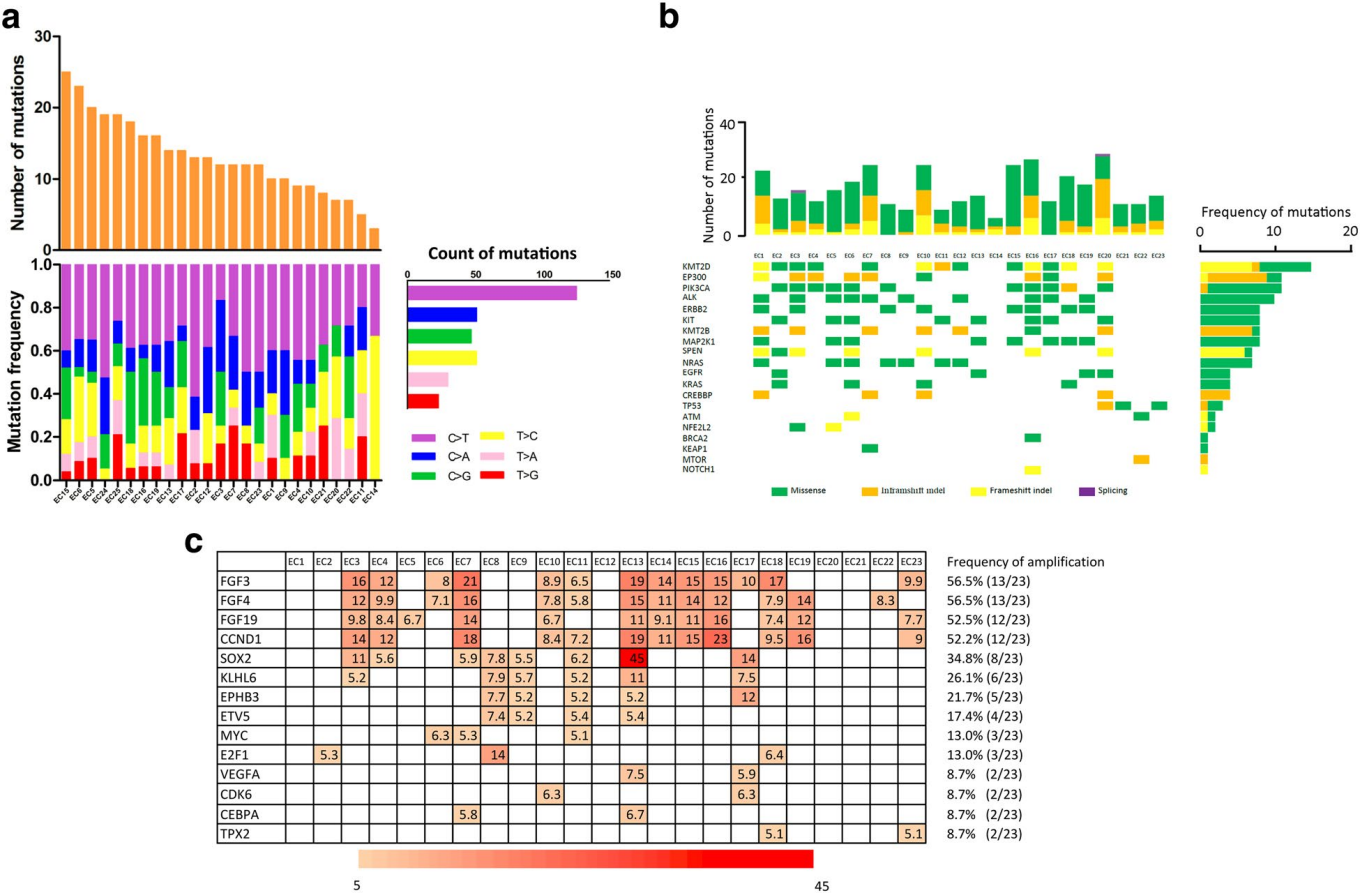
Genomic characterization identified in PDX models of ESCC. **a** Spectrum of somatic point mutations were analyzed and each bar represents one sample. Samples were ordered by the number of mutated genes (top). Six types of single nucleotide transversion were found (different colors; horizontal axis). The vertical axis depicts percent of mutations attributed to a specific mutation type (bottom). Total gene numbers for six classes of base substitutions in 23 PDX models (right). **b** Mutation frequencies and signatures in 23 PDXs of ESCC. Number of somatic mutations of each examined case (top). The ten most frequently mutated genes and ten important genes identified in the literatures, including total mutated samples per gene and mutation subtypes (If multiple mutations were observed within a gene in a single sample, only one was shown. Columns, examined cases; Rows, genes (bottom). Frequencies of somatic mutations for each gene shown on right. **c** Summary of copy number variation. Genomic identification of 14 genes amplified in two or more models. Genomic profile with copy number data (> 5) in heat map. Color bars are a gradient in accordance with number. Frequency of amplification on left

Using next generation sequencing (NGS) to molecularly profile xenografts, we identified 139 mutated genes from a total of 483 genes. Somatic mutations were variable (3–22; Fig. 3a, top). The observed mutation spectrum could be indicative of specific mutagenesis mechanisms occurring in ESCC. Among the 139 single-nucleotide variants, nucleotide transversion was over-represented, particularly C:G > T:A changes (Fig. 3a, bottom and right), which is consistent with ESCC patients in a previous study [27, 28].

Apart from the 139 genes with nonsynonymous single nucleotide variants (SNVs), 79 genes including insertions and deletions (InDels) in protein-coding regions were identified in the 23 PDXs. Thus, 191 non-silent somatic mutated genes were identified, with the range of 6-27 genes for each PDX (Fig. 3b, top). Of which, 96 genes were mutated in 2 or more samples. The top 10 mutated genes (frequency ≥ 7) in the 23 PDX models, and the 10 frequently mutated genes from other reports [27–32] appear in Fig. 3b. KMT2D was the most frequently mutated gene, involving the 7 frameshift inDel, one nonframeshift inDel and 7 missense mutation followed by EP300, PIK3CA, ALK and ERBB2. Frequencies of somatic mutations for each gene appear in Fig. 3b (right).

To interrogate the copy number alteration, 311 genes with copy number alterations (CNAs) were identified (median = 3.96, range 2.8–39.18). According to previous reports, genes with CNAs ≥ 5 are considered amplified [33, 34]. Finally, 63 genes were identified. And, we found 14 genes that were amplified in two or more PDX models (Fig. 3c). FGF3 (13/23), FGF4 (13/23), FGF19 (12/23). CCND1 (12/23) were the most frequently amplified genes and all had an amplification peak spanning 11q13.3. Additional peaks involving important cancer genes such as SOX2, MYC, VEGFA and CDK6 were also found. To demonstrate the amplification, we detected the expression of FGF3, FGF4, FGF19 using quantitative real-time PCR as reported papers [35]. All the samples were classified into two groups (CNA ≥ 5 and CNA < 5) according to the CNA tested by NGS, and CNAs ≥ 5 were considered amplified as it was mentioned above. As was shown in Additional file 2: Figure S1, in agreement with the CNA analysis, the relative mRNA expression of FGF3 (Additional file 2: Figure S1a, p < 0.001), FGF4 (Additional file 2: Figure S1b, p < 0.001) and FGF19 (Additional file 2: Figure S1c, p < 0.05) was enhanced in amplification group (CNA ≥ 5). The results was also in accordance with the previous studies [36, 37], indicating that most of the highly amplified genes showed elevated expression, and the CNAs provided a useful reference for amplification analysis.

**Fig. 4:**
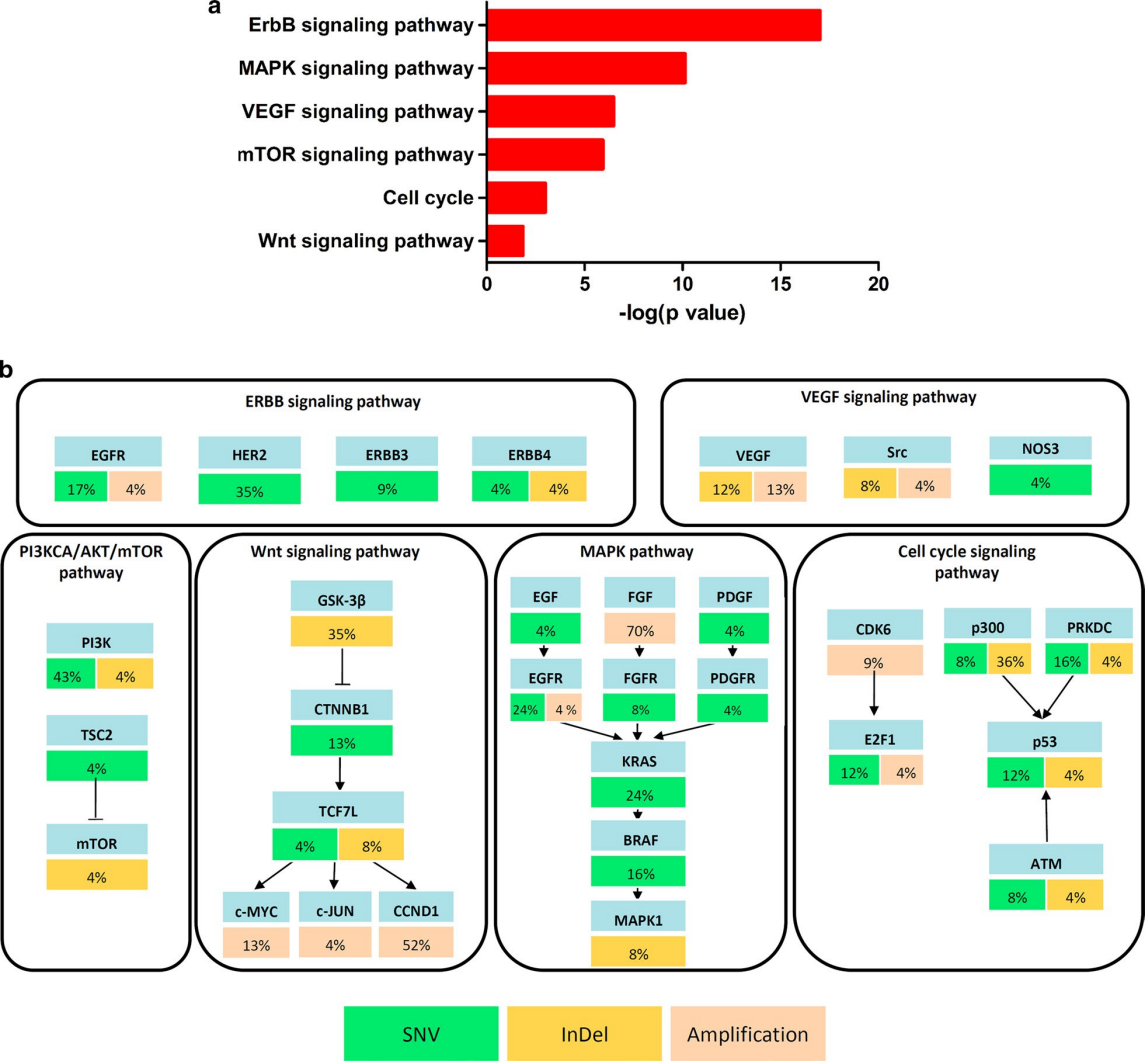
Dysregulated pathways in PDX models of ESCC. **a** Significantly altered pathways (p < 0.05) were enriched and presented. **b** ESCC potential driver genes, mutation frequencies mapped and alteration frequencies shown as percent of all samples; genes identified as SNV are green; InDel are yellow; and amplified genes are pink. Excitatory (arrows) and inhibitory (black lines) interactions were obtained from the KEGG pathway database

Abnormal genes with SNV, InDel or amplifications were analyzed with DAVID Bioinformatics Resources 6.7, and significantly altered pathways (p < 0.05) were enriched and presented (Fig. 4a). The ErbB signaling pathway was the most significantly altered one, followed by the MAPK signaling pathway. Other pathways and abnormal genes appear in Fig. 4b. Most targets and pathways have been reported as candidate targets in carcinomas [7, 38–42]. These results suggested that the PDX models we established may be used for potential target selection and mechanism research.

## Discussion

As an aggressive disease, esophageal cancer is known for limited therapeutic options. So models that enable functional studies translate into the clinic are invaluable. In this study, we have demonstrated that PDX model of esophageal cancer could be derived from very small biopsy specimens. And the histopathological features and chemosensitivity of PDXs were in great accordance with the corresponding primary tumors of patients.

However, the engraftment rate was only 13.3%, which was lower than 34.1% of gastric cancer established using biopsied tissues in our previous reports, and that 34–54% of esophageal cancer in other studies [17, 20, 21, 43]. The difference may result from the following aspects: first, the type of disease. In spite of being gastrointestinal malignant, esophageal cancer is quite different with gastric cancer in the original sites of disease, pathogenesis, genetic profile and so on [44–47], which might lead to variant engraftment rate. Apart from this, the amount of primary tumor tissue might be an important factor influencing the rate of tumor formation. As it is known, it is not easy to get esophageal biopsy specimens for the special and thin anatomical structure. For the limited biopsy tissue, only one site of a mouse can be engrafted for each case. However, the tumor tissues from surgery are always sufficient to be implanted in multiple sites, which may increase the engraftment rate accordingly. Actually, most patients with esophageal cancer are diagnosed in advanced stage, and loss the opportunity of eradicative resection, so biopsy is the only way to establish PDX models. Besides, the paired biopsies before and after chemotherapy could help us to explore the drug-resistance mechanism in the PDX models.

Using our panel of xenograft models, we demonstrated that the model histology correlate well with the primary patient and remained similar between passages. However, some samples had small morphological variations with respect to differentiation perhaps due to the inherent heterogeneity of ESCC and the variability in sampling. ESCC is a type of carcinoma with high intratumoral heterogeneity [30]. Because all PDX models in our study were established with biopsied tissue, only a small fraction of primary tumor can be obtained for implantation, which may different from the specimen for diagnosis. That may explain partially the discrepant differentiation between PDX model and primary tissue. Also, difference might arise from the selection pressures during engraftment in different hosts [48]. Moreover, 5 xenograft models were treated with same chemotherapy agents to patients, and response was in accordance with that of the patients. Our study showed very promising results that the drug sensitivity in PDX assays correlates with patient clinical response, which could provide a realistic model for drug sensitivity selection for ESCC patients.

Pharmacotherapy is the main treatment in advanced ESCC. However, the existing drugs showed little effect in prolonging the overall survival, so it is urgent to develop new agents for these patients. Current approaches of personalized medicine have been incorporating NGS technologies for wide genomic profiling of patient tumors to identify novel therapeutic targets. PDX models may provide a more feasible approach to develop novel agents, study the response to pharmacotherapies and explore predictive markers. The success of targeted therapies in stratifying treatment has underscored the importance of performing mechanistic and functional investigations on breast cancer, NSCLC, colon cancer, gastric cancer and so on [49, 50]. In this study, we report extensive molecular characterization of the 23 ESCC PDX models using NGS technology. The results showed ErbB, MAPK, VEGF, mTOR, cell cycle, and Wnt signaling pathway were mostly frequent abnormal pathways, which were similar to previous reports from ESCC patients [35, 51–53]. However, there is still no effective targeted therapy for ESCC patients. So the labeled mice model corresponding to certain gene or pathway could be available for preclinical evaluation of targeted drug candidates, which will be useful for further application. For example, in our previous study, the anti-tumor effect of CDK4/6 inhibitor SHR6390 was demonstrated in ESCC [54]. And by analyzing the CDK6 expression in esophageal PDX model and transfecting esophageal cancer cell line with small interfering RNA, we found that the CDK6 expressions may be a useful marker to identify the patients who are more likely to benefit from treatment with SHR6390. Apart from it, somatic mutations in the tyrosine-kinase domain of EGFR were identified in 30-50% non-small-cell lung cancers (NSCLCs) patients, among which the TKIs response rate increased to approximately 75% [55, 56]. But the EGFR mutations appear to be a rare studied field in ESCC. In our study, the disrupted ERBB pathway including mutation in ERBB2 (35%), which can be used to explore the TKIs therapy in esophageal cancer. Besides, in a cohort of metastatic renal cell carcinoma patients, mutations in TSC2 were more common in patients who experienced clinical benefit from mTOR inhibitors than in those who progressed [57], indicating that the mutation of TSC2 would be a good prognosis indicator for mTOR inhibitors. But there is no related studies in esophageal cancer, so the PDX models with TSC2 mutation will provided a useful tool.

There are also limitations which should be emphasized. Firstly, the PDX model were established on the NOD/SCID mice, which is lack of integrated immune system. This need to be careful when considering immunological therapies. However, the emerging humanized immune mice model can be provided as a promising model which will solve the problem in a great extent. PDX models can also be a live biobank to offer tumor tissue to establish a more advanced preclinical model which imitates the tumor and immune phenotype partially. Secondly, due to the small volume of the biopsy samples, we could not get the enough samples to validate the molecular characteristic between the PDX and corresponding primary tumor in patients. The establishment of ESCC PDX models in this study was being continued and the rapid improvement in quality, quantity, and cost of NGS will help with this.

## Conclusions

We have shown that PDX models of ESCC can be generated from small biopsy samples and used for functional and genomic studies. It could provide a reliable model for developing new agents and exploring the predictive markers, and therefore provide evidence for individual therapy.

## Additional file

**Additional file 1: Table S1.** The list of 483 cancer-associated genes. **Table S2.** Patient characteristics and transplantation rate. **Table S3.** Evaluation of histology and differentiation between primary patient tumor and the xenografts.
**Additional file 2: Figure S1.** Correlation between mRNA expression and CAN of FGF3 (a), FGF4 (b), and FGF19 (c) in esophageal PDX samples. The graph show mean values for two groups (CNV≥5 or CNV<5). The mRNA expression of the gene of interest was expressed in relation to that of β-actin, used as a housekeeping gene. Error bars represent ± S.E.M. (t-test).

## Abbreviations

PDX: patient-derived xenografts
ESCC: esophageal squamous cell carcinoma
SNV: single nucleotide variant
InDel: insertion and deletion
CNA: copy number alteration
NGS: next generation sequencing
