# Supplementary material for: Establishment and genomic characterizations of patient-derived esophageal squamous cell carcinoma xenograft models using biopsies for treatment optimization
Source: J Transl Med. 2018 Jan 25;16:15. doi: 10.1186/s12967-018-1379-9 (PMC5785825; doi:10.1186/s12967-018-1379-9)
Supplement: Supplementary file 1 — Additional file 1: Table S1. The list of 483 cancer-associated genes. Table S2. Patient characteristics and transplantation rate. Table S3. Evaluation of histology and differentiation between primary patient tumor and the xenografts. [file 12967_2018_1379_MOESM1_ESM.docx]

**Supplementary Table 1. The list of 483 cancer-associated genes**

| ABCB1 | CDK12 | ETV5 | ﻿KAT6A | PAK3 | SMO |
| --- | --- | --- | --- | --- | --- |
| ABCC1 | CDK2 | ETV6 | KDM5A | PALB2 | SOCS1 |
| ABCC2 | CDK4 | EWSR1 | KDM5C | PARP1 | SOD2 |
| ABCC4 | CDK5 | EZH2 | KDM6A | PARP2 | SOX10 |
| ABCC6 | CDK6 | ﻿FAM46C | VEGFR | PAX5 | SOX2 |
| ABCG2 | CDK7 | FANCA | KEAP1 | PBRM1 | SOX9 |
| ABL1 | CDK8 | FANCC | KIT | PDCD1 | SPEN |
| TNK2 | CDK9 | FANCD2 | KLC3 | PDGFRA | SPG7 |
| ACVR1B | CDKN1B | FANCE | KLHL6 | PDGFRB | SPOP |
| AKT1 | CDKN2A | FANCF | KMT2A | PDK1 | SRC |
| AKT2 | CDKN2B | FANCG | KMT2B | PHF6 | SRD5A2 |
| AKT3 | CDKN2C | FANCL | KMT2C | PHKA2 | SRMS |
| ALK | CEBPA | FBXW7 | KMT2D | PIGF | STAG2 |
| AMER1 | CHEK1 | FCGR3A | KRAS | PIK3CA | STAT1 |
| APC | CHEK2 | FGF10 | ﻿LCK | PIK3CB | STAT2 |
| AR | CHST3 | FGF14 | LIMK1 | PIK3CG | STAT3 |
| ARAF | CIC | FGF19 | LMO1 | PIK3R1 | STAT4 |
| ARFRP1 | COMT | FGF23 | LRP1B | PIK3R2 | STAT5A |
| ARID1A | CREBBP | FGF3 | LRP2 | PRRT2 | STAT5B |
| ARID1B | CRKL | FGF4 | LYN | PRKCG | STAT6 |
| ARID2 | CRLF2 | FGF6 | ﻿MAP2K1 | PRKCE | STEAP1 |
| ASXL1 | CSF1R | FGFR1 | MAP2K2 | PLK1 | STK11 |
| ATIC | CSK | FGFR2 | MAP2K4 | PPARD | STK3 |
| ATM | CSNK1A1 | FGFR3 | MAP3K1 | PPP1R13L | STK4 |
| ATP7A | CTCF | FGFR4 | MAP4K4 | PPP2R1A | SUFU |
| ATR | CTLA4 | FGR | MAP4K5 | PRDM1 | SULT1A1 |
| ATRX | CTNNA1 | FKBP1A | MAPK1 | PRDX4 | SULT1A2 |
| AURKA | CTNNB1 | FLT1 | MAPK10 | PRKAA1 | SULT1C4 |
| AURKB | CYBA | FLT3 | MAPK14 | PRKAR1A | SYK |
| AXIN1 | CYLD | FLT4 | MAPK8 | PRKCA | ﻿TCF7L1 |
| AXL | CYP19A1 | FOXL2 | MAPK9 | PRKCB | TCF7L2 |
| ﻿B2M | CYP1A1 | FRK | MAPKAPK2 | PRKDC | TEK |
| BAIAP3 | CYP1A2 | FUBP1 | MARK1 | PTCH1 | TET2 |
| BAP1 | CYP1B1 | FYN | MCL1 | PTEN | TGFBR1 |
| BARD1 | CYP2A6 | FZD7 | MDM2 | PTK2 | TGFBR2 |
| BCL2 | CYP2B6 | GALNT14 | MDM4 | PTPN11 | TK1 |
| BCL2L2 | CYP2C19 | GATA1 | MED12 | PTPRD | TMPRSS2 |
| BCL6 | CYP2C8 | GATA2 | MEF2B | ﻿RAC2 | TNF |
| BCOR | CYP2C9 | GATA3 | MEN1 | RAD50 | TNFAIP3 |
| BCORL1 | CYP2D6 | GCK | MERTK | RAD51 | TNFRSF14 |
| BCR | CYP2E1 | GID4 | MET | RAF1 | TNFRSF8 |
| BIRC5 | CYP3A4 | GINS2 | MITF | RARA | TNFSF11 |
| BLK | CYP3A5 | GNA11 | MKNK2 | RB1 | TNFSF13B |
| BLM | CYP4B1 | GNA13 | MLH1 | RET | TOP1 |
| BRAF | ﻿DAXX | GNAQ | MPL | RICTOR | TP53 |
| BRCA1 | DDR1 | GNAS | MRE11A | RMDN2 | TPMT |
| BRCA2 | DDR2 | GPC3 | MS4A1 | RNF43 | TPX2 |
| BRIP1 | DNMT1 | GPR124 | MSH2 | ROCK1 | TRAIL-R1 |
| PTK6 | DNMT3A | GRIN2A | MSH6 | MST1R | TRAIL-R2 |
| CD147 | DOT1L | GSK3B | MTDH | ROS1 | TSC1 |
| BTK | DPYD | GSTM1 | MTHFR | RPL13 | TSC2 |
| ﻿C11orf30 | DSCAM | GSTM3 | MTOR | RPS6KA1 | TSHR |
| C18orf56 | ﻿E2F1 | GSTP1 | MTRR | RPS6KB1 | TYMS/TS |
| C8orf34 | EGF | ﻿GSTT1 | MUTYH | RPTOR | TYRO3 |
| CAMK2G | EGFL7 | H3F3A | MYC | RRM1 | ﻿U2AF1 |
| CAMKK2 | EGFR | ﻿HCK | MYCL1 | RUNX1 | UBE2I |
| CARD11 | EGR1 | HGF | MYCN | ﻿SCF/KITLG | UGT1A1 |
| CASP8 | EMC8 | HIF1A | MYD88 | SDHA | UGT1A9 |
| CBFB | EML4 | HIST1H3B | ﻿NAT1 | SDHAF1 | UGT2B15 |
| CBL | ENOSF1 | HNF1A | NAT2 | SDHAF2 | UGT2B17 |
| CBR1 | EP300 | HRAS | NCAM1 | SDHB | UGT2B7 |
| CBR3 | EPHA1 | HSP90AA1 | NCF4 | SDHC | UMPS |
| CCND1 | EPHA2 | ﻿IDH1 | NCOA3 | SDHD | ﻿VEGFA |
| CCND2 | EPHA3 | IDH2 | NCOR1 | SETD2 | VEGFB |
| CCND3 | EPHA4 | IGF1 | NEK11 | SF3B1 | VHL |
| CCNE1 | EPHA5 | IGFR | NF1 | SGK1 | ﻿WEE1 |
| CCR4 | EPHA7 | IGF2 | NF2 | SHH | WISP3 |
| CD19 | EPHA8 | IGF2R | NFE2L2 | SIK1 | WNK3 |
| CD22 | EPHB1 | IKBKB | NFKBIA | SKP2 | WT1 |
| CD274 | EPHB2 | IKBKE | NKX2-1 | SLC10A2 | ﻿XPC |
| CD33 | EPHB3 | IKZF1 | NOS3 | SLC15A2 | XPO1 |
| CD38 | EPHX1 | IL7R | NOTCH1 | SLC22A1 | XRCC1 |
| CD3EAP | ERBB2 | INHBA | NOTCH2 | SLC22A16 | XRCC4 |
| CD52 | ERBB3 | INSR/IR | NPM1 | SLC22A2 | YES1 |
| CD74 | ERBB4 | IRF4 | NQO1 | SLC22A6 | ZAP70 |
| CD79A | ERCC1 | IRS2 | NRAS | SLCO1B1 | ZC3HAV1 |
| CD79B | ERCC2 | ITK | NTRK1 | SLCO1B3 | ZNF217 |
| CDA | ERG | ﻿JAK1 | NTRK2 | SMAD2 | ZNF703 |
| CDC73 | ESR1 | JAK2 | NTRK3 | SMAD4 |  |
| CDH1 | ETV1 | JAK3 | NUP93 | SMARCA4 |  |
| CDK1 | ETV4 | JUN | ﻿PAK1 | SMARCB1 |  |

**Supplementary Table 2. Patient characteristics and transplantation rate**

| Characteristics | No. of patients (%) | Transplantation rate (%) | *p* value |
| --- | --- | --- | --- |
| Gender |  |  |  |
| Male | 169 | 14.8% (25/169) | 0.082 |
| Female | 19 | 0 (0/19) |  |
| Age (years) |  |  | 0.073 |
| ≥60 | 99 | 9.1% (9/99) |  |
| <60 | 89 | 18 % (16/89) |  |
| Primary Location |  |  | 0.97 |
| Upper | 17 | 11.8% (2/17) |  |
| Median | 83 | 13.3% (11/83) |  |
| Lower  NA | 80  8 | 13.8% (11/80)  12.5% (1/8) |  |
| Histology |  |  | 0.54 |
| Adenocarcinoma | 13 | 15.4% (2/13) |  |
| Squamous carcinoma | 175 | 13.1%(23/175) | 0.07 |
| Well differentiation | 11 | 0 (0/11) |  |
| Moderate differentiation | 96 | 13.7% (13/96) |  |
| Poor differentiation  NA | 51  17 | 16.2% (10/51)  0 (0/17) |  |
| Stage |  |  |  |
| I~II | 19 | 0 (0/19) |  |
| III~IV  NA | 124  45 | 17.7% (22/124)  6.7% (3/45) | 0.045 |
| Chemotherapy |  |  |  |
| Before | 129 | 14.8% (19/129) |  |
| After | 42 | 7.1% (3/42) | 0.29 |
| NA | 17 | 17.6%(3/17) |  |

Note: NA, non-available. *p value* calculated by unpaired two-tailed t-test or one-way analysis of variance separately.

**Supplementary table 3**

**Evaluation of histology and differentiation between primary patient tumor and the xenografts**

|  |  | Primary tumor | | P3 | P4 | Concordance rate (%) |
| --- | --- | --- | --- | --- | --- | --- |
| Histology |  | |  |  |  | 100 (25/25) |
|  | Adenocarcinoma | | 2 | 2 | 2 |  |
|  | Squamous carcinoma | | 23 | 23 | 23 |  |
| Differentiation |  | |  |  |  | 64(16/25) |
|  | Well | | 0 | 0 | 1 |  |
|  | moderate | | 15 | 11 | 8 |  |
|  | Poor | | 10 | 14 | 16 |  |
