# Supplementary figures and images for: Establishment and genomic characterizations of patient-derived esophageal squamous cell carcinoma xenograft models using biopsies for treatment optimization
Source: J Transl Med. 2018 Jan 25;16:15. doi: 10.1186/s12967-018-1379-9 (PMC5785825; doi:10.1186/s12967-018-1379-9)

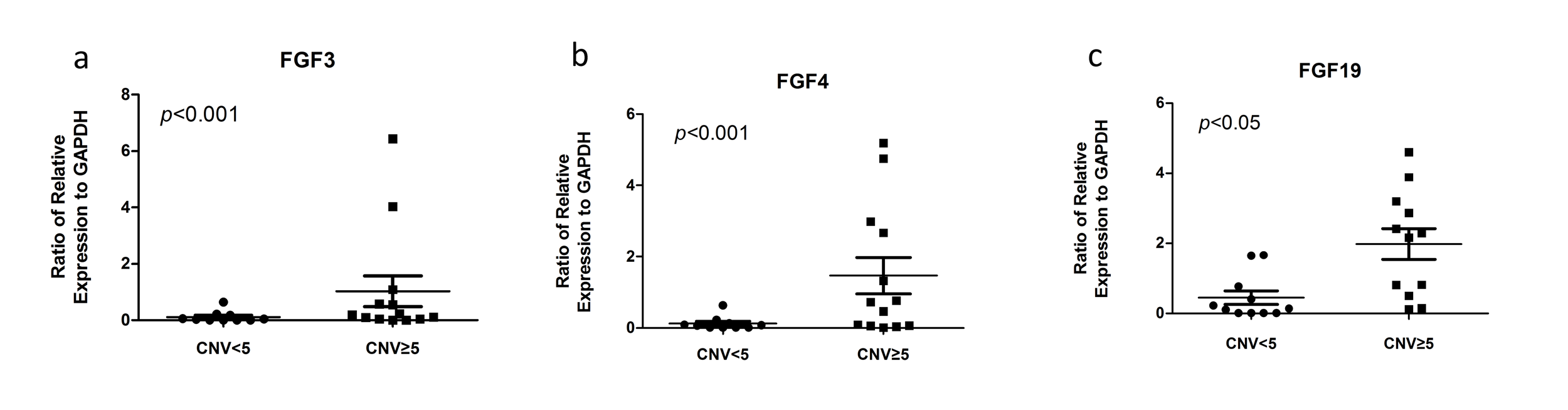

Supplement: Supplementary file 2 — Additional file 2: Figure S1. Correlation between mRNA expression and CAN of FGF3 (a), FGF4 (b), and FGF19 (c) in esophageal PDX samples. The graph show mean values for two groups (CNV≥5 or CNV<5). The mRNA expression of the gene of interest was expressed in relation to that of β-actin, used as a housekeeping gene. Error bars represent ± S.E.M. (t-test). [file 12967_2018_1379_MOESM2_ESM.tif]
